# The relationship between social support, coping strategies and psychological distress and positive mental well-being in carers of people with borderline personality disorder

**DOI:** 10.1186/s40479-023-00237-w

**Published:** 2023-10-12

**Authors:** Aoife Hayes, Maria Dempsey, Mary Kells, Mike Murphy

**Affiliations:** 1 School of Applied Psychology, University College Cork, Cork, Ireland; 2 Mental Health Services, Cork Kerry Community Healthcare, Health Service Executive, Cork, Ireland

**Keywords:** Borderline personality disorder, Carers, Coping, Social support, Psychological distress, Well-being

## Abstract

**Background:**

Informal carers of people with BPD experience high levels of burden and psychological distress relative to other populations. There is a scarcity of research evidencing the influence of modifiable factors on carer outcomes to inform interventions. This study aimed to investigate the relationship between social support, coping strategies and psychological distress and positive mental well-being in this carer population.

**Methods:**

In this cross-sectional study, 1207 carers completed the McLean Screening Instrument for BPD-Carer Version, the Brief COPE, the Multidimensional Scale of Perceived Social Support, the Kessler Psychological Distress scale, the WHO-5 Well-being Index, and the Coronavirus Anxiety Scale. Data for 863 participants who met the inclusion criteria were analysed.

**Results:**

Carers reported low positive mental well-being and high psychological distress. Perceived social support and several coping strategies were significant unique predictors of psychological distress and positive mental well-being. Perceived social support and positive reframing were the strongest predictors of higher positive mental well-being and lower psychological distress. Self-blame, behavioural disengagement and substance use were the strongest predictors of adverse outcomes.

**Conclusions:**

The findings evidence modifiable factors that may be used to improve informal carer outcomes and indicate that carer interventions may be improved by focusing on reducing the use of self-blame, behavioural disengagement and substance use, and development of quality social support and skills to positively reframe caregiving situations.

## Introduction

Borderline Personality Disorder (BPD) is characterised by persistent dysregulation in emotions, relationships with others, self-image, and by behavioural impulsivity [1]. An estimated 75% of people with BPD have self-harmed [2]; an estimated 10% complete suicide [3]. In many countries community-based treatment is now recommended, with limited admission to non-specialist inpatient services [4, 5]. As a result, family members and significant others - informal carers (ICs) - are at the frontline of informal support for individuals with BPD in the community [6–8].

In interviews, ICs of people with BPD have reported traumatic stress, exhaustion, feelings of hopelessness, worry, guilt, shame, sadness and despair [8–11] and that being an IC alters their lives; may limit engagement in their other life roles and access to social support [11–13]; and may contribute to increased inter-family relationship strain [8–10] and neglect of personal well-being and self-care [10, 12]. Significantly higher levels of burden and grief are reported by ICs of people with BPD than by ICs of people with other mental health diagnoses, and greater levels of psychological distress are found compared to population norms [2, 14–17].

National guidelines [4, 5] recognise challenges faced by, and potential support needs of, ICs of people with BPD. Several interventions exist. Family Connections is based on a biosocial model of BPD and Dialectical Behaviour Therapy [18]. Effectiveness studies have shown pre- to post- reduction in burden and grief and increased mastery [5, 17] and family functioning [19]. However, compared to optimised treatment-as-usual, only burden and grief had a significant treatment effect [20]. No randomised controlled trial (RCT) has yet been published. Mentalization Based Therapy-Families and Carers Training and Support is based on mentalization-based treatment for BPD [21]. A delayed treatment RCT revealed post-treatment improvements in carer anxiety, distress, burden, mastery and family functioning and a reduction in adverse incidents with the person with BPD. However compared to waitlist control, post-treatment effects were only found for adverse incidents, well-being and family functioning [21]. Staying Connected is informed by a relational model of personality disorders [22]. A RCT found no pre-to-post change in IC distress or burden; however significant pre- to post-treatment improvements in measures of family interaction were found compared to waitlist. Further, there was also a 12 month improvement in IC distress and burden, though long-term outcomes were not compared to control [23]. Making Sense of BPD is informed by Cognitive Analytical Therapy and is an element of the Helping Young People Early programme for young people with BPD characteristics [24]. A pilot study found reduced subjective burden and increased personality disorder knowledge; no RCT has yet been published. While the extant research indicates emerging positive effects of existing interventions, modifications may improve IC outcomes. Research to identify modifiable factors associated with better carer outcomes may help to inform refinement and potentially enhance treatment efficacy [25, 26].

However, there remains a paucity of research on factors predicting outcomes for this carer population. To date, research has reported that being a female IC [2, 16], difficulties with emotion regulation [14, 15], emotional overinvolvement (e.g. overconcern or overidentification with the person with BPD), criticism (e.g. critical comments about the person with BPD) [2, 14], and higher frequency of self-harm and suicide attempts by the person with BPD [14] correlate with higher carer burden and distress. The relationship between carer age and outcome is inconsistent [2, 14, 16]. Parents and spouses/partners do not differ in levels of reported burden or psychological distress [2, 14, 16].

Stress process models explain carer outcomes in response to caregiving stress [27] and propose that social support and coping strategies are important moderators of IC adjustment across carer populations [28–33]. There is evidence of applicability of the model in ICs of people with personality disorders [34].

Coping is the process of behaviourally and cognitively responding to a situation appraised as stressful [35]. In other IC populations, the use of coping strategies predicted carer burden and mental health (e.g. 32, 36). Maladaptive coping strategies predicted psychological distress in ICs of people with eating disorders [37] and affective disorders [33]. Avoidance coping strategies correlated with burden in ICs of people with schizophrenia and bipolar affective disorder [38]. In carers of people with schizophrenia, coping strategies of self-blame, collusion and criticism related to adverse outcomes; whereas strategies of positive reappraisal, reframing and religious coping correlated with better well-being in more than one study [39–44]. Higher levels of social support relate to better outcomes across IC populations, including ICs of people with mental health diagnoses [34, 45]. Unfortunately, caregiving can be associated with lower perceived social support [46], as revealed by small samples of ICs of people with BPD [10–13].

To date, research has focused on the presence or absence of negative IC outcomes, such as grief, burden and psychological distress and has not included measures of presence of positive well-being. Good mental health or well-being requires the presence of positive psychological well-being, extending beyond the absence of illness and distress [47–50]. Factors influencing positive well-being differ from those influencing distress [47, 48] and some health outcomes, for example longevity [51], are predicted by the presence of positive well-being rather than the absence of illness or distress. Relative to positively worded psychological well-being scales, such as the WHO-5, ceiling effects can be found when measures include negatively worded depressive symptoms [50]. Therefore it is important that studies assess the relationship between possible moderating factors and both negative carer outcomes (such as psychological distress) and positive outcomes (such as positive mental well-being, measured using positively worded items).

The extant research has established that ICs of people with BPD may experience significant burden and distress. However negative caregiving effects are not inevitable. It is important to identify factors associated with better outcomes to develop an empirical evidence base that clinicians and others can use to help optimise interventions, with the aim of better supporting this caregiving population. This study aims to examine the relationship between perceived social support and coping and the outcomes of psychological distress and positive mental well-being in ICs of people with BPD.

## Method

### Design and procedure

In this cross-sectional study, ICs of people with BPD completed a self-report online survey advertised on a public mental health service website and an international community forum and sent via email to people registered with two international organisations supporting ICs of people with BPD and to registered past and current attendees of a local programme for ICs of people with BPD. The online survey comprised the following sections: [1] information about the study and request to consent to participate; [2] a sociodemographic questionnaire; [3] the measures described below; and [4] four open-ended qualitative questions related to COVID-19. This empirical paper focuses on the quantitative data. After securing institutional ethical approval, recruitment occurred between August and November 2020.

### Participants

Participants were self-selecting individuals who identified as ICs of a person with BPD. Participant inclusion criteria were: [1] minimum 18 years of age; [2] English-speaker; [3] a relative or significant other of a person with BPD/ Emotionally Unstable Personality Disorder (EUPD); [4] supporting or caring for a person with BPD/ EUPD; and [5] endorse a statement that the person they care for has BPD/ EUPD. Respondents were screened for inclusion criteria [1] to [5] at the beginning of the survey. Participants were included in the current study if they were caring for a person with BPD/ EUPD who was minimum 12 years of age and identified as the primary carer. Therefore, in the current research, the term IC refers to a person who identified as the primary carer and could be a family member or significant other (e.g. friend) of a person with BPD, with no limits on the type of care provided, cohabitation or amount of contact.

### Measures

A sociodemographic questionnaire comprised questions about the IC, the person with BPD, and the caregiving relationship (see Table 1).

**Table 1:** Characteristics of the Informal Carer and the Person with BPD (n = 863)

Carer			
Age (n = 856)		Mean (SD)	53.5 (10.7)
Gender (n = 862)		Male Female Non-binary Prefer not to say	118 (13.7%) 739 (85.7%) 3 (0.3%) 2 (0.2%)
Education level (n = 862)		Less than graduate degree Graduate degree Postgraduate degree	239 (27.7%) 314 (36.4%) 309 (35.9%)
Employment		Working Not working	586 (67.9%) 277 (32.1%)
Relationship to person with BPD		Parent Child Sibling Spouse/ Partner Significant other	649 (75.2%) 32 (3.7%) 19 (2.2%) 137 (15.9%) 26 (3.0%)
Living with the person with BPD		Yes	521 (60.4%)
Mean years caregiving ^1^		Mean (SD)	12.7 (9.8)
Mean hours contact per week in last month		Mean (SD)	49.6 (54.5)
Received intervention^2^		Yes	639 (74%)
Chronic illness		Yes	244 (28.3%)
Person with BPD			
Age (n = 862)		Mean (SD)	29.8 (13.5)
Gender (n = 861)		Male Female Non-binary Prefer not to say	191 (22.1%) 640 (74.2%) 25 (2.9%) 5 (0.6%)
Employment (n = 862)		Working Not working	265 (30.7%) 597 (69.2%)
Estimated no. days self-harm of the PwBPD in last year (n = 615)^3^		Mean (SD)	22.5 (59.8)
Estimated no. of days attempted suicide in last year (n = 772)^3^		Mean (SD)	1.0 (4.4)

Note: n = number of people who responded. 1 Two extreme outliers (100, 80) replaced with the highest value that is not an outlier (49 years). 2. The respondent was asked if they had received any intervention, education, or supports to guide them in their role as a carer for a person with BPD. 3. These values were reported by the IC rather than the PwBPD. The survey asked IC participants if they knew or were estimating the number of days of reported self-harm and attempted suicide days of the PwBPD. 19.5% of ICs that responded to the question on days of self-harm reported that they knew the number of days of self-harm by the PwBPD in the last year; 80.5% of ICs reported that their response was an estimate. 50.5% of ICs that responded to the question on days of attempted suicide reported that they knew the number of days of attempted suicide by the PwBPD in the last year; 49.5% said their response was an estimate

The McLean Screening Instrument for Borderline Personality Disorder – Carer Version (MSI-BPD-C) is a 10-item screening instrument, adapted by Goodman et al. [52] from the MSI-BPD [53], to allow ICs to report on symptoms of the person with BPD and used to validate participants’ self-report that the person they are caring for has a BPD diagnosis [2, 14, 15, 23]. Endorsement of seven items or more on the MSI-BPD is reported to have an acceptable diagnostic accuracy (AUC = 0.73-0.83) and test-retest reliability (ICC = 0.72–0.78) [53–54]. A mean score of 8.6 (SD = 1.05) was found in the current sample. Inclusion criterion for the current study was a minimum score of seven on the MSI-BPD-C.

The Brief COPE [55], adapted from the Coping Orientation to Problems Experienced (COPE) Inventory [56], includes 28 statements about coping methods, comprising 14 subscales (Table 2), and has previously been used in IC populations [57]. The subscales have demonstrated Cronbach’s alpha between 0.5 and 0.9 [55]. The developers recommend a study-specific assessment of the factor structure. Wording can be adapted to specify situational coping, particular to the focus of research [55]. In this research, participants were asked what they were “doing to deal with the stress associated with caring for a person with Borderline Personality Disorder/ Emotionally Unstable Personality Disorder”. The original 14 subscales were retained in the analysis. Mean subscale inter-item correlations between 0.27 and 0.93 indicated adequate internal consistency [58].

**Table 2:** Descriptive Statistics of Self-report Measures

	Mean	SD
Psychological distress (K10)	24.99	7.74
Positive mental well-being (WHO-5)	42.01	20.89
Coping strategies		
Self-distraction	5.79	1.51
Active coping	5.73	1.63
Denial	2.59	1.16
Substance use	3.22	1.77
Use emotional support	5.05	1.77
Use instrumental support	5.01	1.80
Behavioural disengagement	3.43	1.57
Venting	4.29	1.38
Positive reframing	4.72	1.64
Planning	6.09	1.64
Humour	3.16	1.46
Acceptance	6.37	1.46
Religion	4.50	2.17
Self-blame	4.62	1.89
Perceived social support (MSPSS)	4.59	1.40
Coronavirus (COVID-19) anxiety	1.76	2.97

The Kessler Psychological Distress scale (K10) [59] measures generalised psychological distress. Participants respond to ten questions about how they’ve been feeling over the last 30 days on a Likert scale from 1 (none of the time) to 5 (all of the time). Total scores range from 10 to 50. The K10 has demonstrated good internal consistency (0.93) [59] and validity when correlated with the General Health Questionnaire (ρ = 0.5) and Short Form-12 (ρ =-0.6) [60]. Scores over 22 represent high levels of psychological distress [61]. It has been implemented in general population research and mental health ICs [17, 43, 60, 62]. Internal consistency in this study was 0.91.

The World Health Organisation – Five Well-being Index (WHO-5), measuring positive mental well-being, is an established measure of subjective psychological well-being [63]. Participants rate frequency of five positive experiences in the past fortnight on a six-point Likert scale from 0 (at no time) to 5 (all of the time). Responses are summed and transformed to give a total score range from 0 to 100. Higher scores show greater levels of positive mental well-being. The WHO-5 is reported to have high content validity [64], construct validity (e.g. r=-.49 compared to Beck’s Depression Inventory, BDI-6 [65]) and adequate reliability (internal consistency 0.84–0.89) [50, 66]. The internal consistency in this sample was 0.89.

The Multidimensional Scale of Perceived Social Support (MSPSS) [67] measures perceived social support. Respondents answer 12 questions on a Likert scale from 1 (very strongly disagree) to 7 (very strongly agree). Adequate internal reliability (0.91–0.92) and one-month test-retest reliability (ICC = 0.90) have been reported. Factorial and construct validity have been demonstrated. The MSPSS score correlates with depression (r=-.48) in the expected direction [68, 69]. The internal consistency in the present study was 0.94.

The Coronavirus Anxiety Scale (CAS) [70] measures anxiety due to COVID-19. Participants respond to five statements, concerning their COVID-19 pandemic experiences over the last fortnight, on a Likert scale from 0 (not at all) to 4 (nearly every day). A higher score represents greater COVID-19 anxiety. The internal reliability is 0.92–0.93, with construct validity illustrated through correlation with measures of impairment, psychological distress and worry about coronavirus [70, 71]. The internal consistency in this study was 0.85.

### Statistical analysis

Data were analysed using IBM SPSS Statistics 26.0 for Windows [72]. Data were missing for 6.6% of participants; 21 people (1.7%) had more than 40% missing data on at least one measure. These participants were excluded from further analysis. Multiple imputation was employed to manage the remaining missing data, which exceeded 5% missingness [73].

Bivariate analyses of the relationship between the sociodemographic variables and the two dependent variables were conducted using correlations, one-way analysis of variance (ANOVAs) and independent sample t-tests. Where a variable was found to be non-normally distributed, non-parametric equivalents were conducted. Parametric test results are reported unless there was a discrepancy with the nonparametric results. Psychological distress and positive mental well-being were compared to published data using one-sample t-tests.

A series of hierarchical multiple regression models investigated the relationship between perceived social support, coping and the dependent variables. Sociodemographic variables that showed a significant bivariate relationship with the dependent variables were included in the regression model as controls. As data for this study was collected during the COVID-19 pandemic, COVID-19 anxiety, measured using the CAS, was included as a control.

Step 1 of each hierarchical regression included the sociodemographic control variables, COVID-19 anxiety and perceived social support. The 14 coping strategies were introduced in step 2 of each model to identify (i) the percentage of the variance explained by the coping strategies combined and (ii) which coping strategies were the strongest predictors.

## Results

Data for 863 of the 1207 participants who completed surveys were analysed. Three hundred and forty-four participants were excluded because the MSI-BPD-C score was less than seven (n = 216), they were not primary carers (n = 105), they were caring for a person with BPD under the age of 12 (n = 2), or there was more than 40% missing data on any one measure (n = 21).

### Descriptive statistics

Tables 1 and 2 detail the descriptive statistics for the sociodemographic characteristics and the self-report measures. The majority of ICs resided in North America (n = 774; 89.6%).

### IC psychological distress and positive mental well-being

Mean psychological distress (M = 24.99, SD = 7.74) was significantly higher than reported in a general adult population (M = 14.5, SD = 9.4) [61], t(862) = 39.82, p < .001, d = 1.35, with a large effect size, and compared to reported values for ICs of people with schizophrenia in China (M = 19.54, SD = 5.29) [62], t(862) = 20.70, p < .001, g = 0.73 and Malaysia (M = 17.8, SD = 7.08) [43], t(862) = 27.30, p < .001, g = 0.94, with a medium to large effect size. The score was similar to that reported for ICs of outpatient adolescents with borderline personality features (M = 24.99, SD = 8.83) [17], t(862) = 0.03, p = .98, g < 0.001. Nearly two-thirds (63%) of the sample had a minimum score of 22, indicating high levels of psychological distress.

Positive mental well-being in the current sample (M = 42.01, 20.89) was significantly lower, with a medium effect size, compared to the mean population score reported for Serbia, the country with the lowest average score on the WHO-5 in the European Quality of Life Survey (EQLS) 2015 (M = 52), t(862)=-14.05, p < .001, d = 0.48 [74], compared to carers in the EQLS (M = 60.84, SD = 22.6) [66], t(862)=-26.47, p < .001, g = 0.84, with a large effect size, and ICs of people with traumatic brain injury (transformed M = 49.6, SD = 24.4), t(862)=-10.67, p < .001, g = 0.36 [75], with a small effect size. Nearly two-thirds (62.6%) of the sample scored less than 50 on the WHO-5 well-being scale, the screening cut-off for depression [63].

### Predictors of psychological distress

Table 3 details the relationship between psychological distress and the sociodemographic variables. Psychological distress was significantly higher for ICs who had undergone postgraduate education than those who had not, t(547) = 3.18, p < .01, g = 0.28. Parents, t(861)=-2.64, p = .008, g = 0.21, had significantly lower and spouses/ partners, t(861) = 3.43, p = .001, g = 0.32, significantly higher distress than the other relationship categories combined, with small effect sizes. Step 1 of the regression included the following: IC age; whether the IC was a spouse/partner, had less than graduate education, was working, reported receiving an intervention/ supports related to their caregiving role, had a chronic illness, or was living with the person with BPD; hours of contact per week with the person with BPD; COVID-19 anxiety; and perceived social support.

**Table 3:** Sociodemographic Variables relationship with Psychological Distress and Positive Mental Well-being

		Psychological Distress			Positive Mental well-being		
	Test	Statistic	p	Effect size^6^	Statistic	p	Effect size^6^
Carer variables							
Age^1,4^	r	− 0.21	< 0.001		0.14	< 0.001	
Gender^2^	F(3, 859)	1.60	0.19	< 0.01	1.19	0.31	< 0.01
Education^2^	F(2, 860)	5.03	< 0.01	0.01	4.45	0.01	0.01
Employment^3^	t(861)	2.22	0.03	0.16	-1.21	0.23	0.09
Intervention^3^	t(861)	6.34	< 0.001	0.49	-4.46	< 0.001	0.35
Chronic Illness^3^	t(861)	-6.23	< 0.001	0.50	6.55	< 0.001	0.50
Person with BPD variables							
Age^1^	r	− 0.02	0.50		0.07	0.05	
Gender^2^	F(3, 859)	1.61	0.19	< 0.01	1.41	0.24	< 0.01
Employment^3^	t(861)	-1.36	0.17	0.10	1.98	0.05	0.15
Estimated no. of days of self-harm^4^	r	0.15	< 0.001		− 0.15	< 0.001	
Estimated no. of days of suicide attempts^4^	r	0.15	< 0.001		− 0.09	0.01	
Relationship variables							
Relation to person with BPD^2^	F(4,858)	3.12	0.02	0.01	2.52	0.04	0.01
Living with person with BPD^3^	t(861)	-4.15	< 0.001	0.28	5.95	< 0.001	0.41
Hours of contact^4^	r	0.17	< 0.001		− 0.23	< 0.001	
Years of caregiving^4^	r	− 0.07	0.05		0.06	0.08	

Note: 1 Pearson correlation; 2 One-way ANOVA 3. T-test. 4 Spearman correlation; 5 Mann-Whitney test; 6 Effect sizes reflect Hedge’s g for t-tests (small (0.2), medium (0.5) and large (0.8)) and η^2^ for one-way ANOVAs (small (0.02), medium (0.13) and large (0.26))

Table 4 summarises the regression results. In step 1, the sociodemographic variables, COVID-19 anxiety, and perceived social support contributed significantly to the regression model, R^2^ = 0.28, F(10, 852) = 33.80, p < .001. The addition of coping strategies in step 2 uniquely explained an additional 21% of the variance. This change was significant with a large effect size when controlling for IC age, education, relationship category, working status, receipt of intervention, chronic illness, hours of contact, COVID-19 anxiety and perceived social support, ΔR^2^ = 0.21, ΔF(14,838) = 25.14, p < .001, f^2^ = 0.43. In the regression model, age, receipt of intervention, and chronic illness were the only significant sociodemographic predictors of distress. COVID-19 anxiety also positively predicted distress. Social support and positive reframing were significant negative predictors of psychological distress. Self-blame, behavioural disengagement, substance use and denial were significant positive predictors of distress. Self-blame and social support were the strongest predictors of psychological distress, uniquely explaining 5.5% and 2.7% of the variance, respectively. Positive reframing, behavioural disengagement and substance use significantly explained 1.6%, 1.3% and 1.2% of the variance, respectively. Denial had little influence on psychological distress, explaining 0.8% of the variance in psychological distress.

**Table 4:** Hierarchical Regression Model of Predictors of Informal Carer Psychological Distress

			B (95% CI)				
	B	SE B	Lower	Higher	β	t	p
Step 1							
(Constant)	38.15	1.69	34.84	41.46		22.60	< 0.001
Age	-0.11	0.02	-0.16	-0.06	-0.15	-4.66	< 0.001
Education (less than graduate)	0.67	0.51	-0.34	1.67	0.04	1.30	0.19
Relationship (spouse/ partner)	-0.09	0.69	-1.43	1.26	0.00	-0.13	0.90
Working	-0.67	0.51	-1.66	0.32	-0.04	-1.32	0.19
Received intervention	-2.00	0.54	-3.06	-0.94	-0.11	-3.71	< 0.001
Chronic illness	2.38	0.51	1.37	3.39	0.14	4.64	< 0.001
Cohabitation	0.96	0.55	-0.13	2.04	0.06	1.72	0.09
Hours of contact	0.00	0.01	-0.01	0.01	0.02	0.43	0.67
COVID-19 anxiety	0.65	0.08	0.50	0.81	0.25	8.46	< 0.001
Perceived social support	-1.63	0.17	-1.96	-1.30	-0.29	-9.73	< 0.001
Step 2							
(Constant)	23.34	2.15	19.12	27.56		10.85	< 0.001
Age	-0.08	0.02	-0.12	-0.04	-0.11	-3.79	< 0.001
Education (less than graduate)	0.20	0.44	-0.67	1.06	0.01	0.45	0.65
Relationship (spouse/ partner)	-0.29	0.61	-1.48	0.91	-0.01	-0.47	0.64
Working	-0.58	0.43	-1.43	0.27	-0.04	-1.35	0.18
Received intervention	-1.06	0.47	-1.99	-0.13	-0.06	-2.24	0.03
Chronic illness	2.16	0.44	1.30	3.02	0.13	4.91	< 0.001
Cohabitation	0.38	0.47	-0.55	1.31	0.02	0.81	0.42
Hours of contact	0.01	0.00	0.00	0.01	0.04	1.22	0.22
COVID-19 anxiety	0.44	0.07	0.30	0.57	0.17	6.45	< 0.001
Perceived social support	-1.15	0.17	-1.48	-0.81	-0.21	-6.74	< 0.001
Self-distraction	0.20	0.13	-0.06	0.46	0.04	1.48	0.14
Active coping	0.19	0.16	-0.12	0.50	0.04	1.22	0.22
Denial	0.69	0.19	0.32	1.06	0.10	3.68	< 0.001
Substance use	0.50	0.11	0.28	0.73	0.12	4.45	< 0.001
Use emotional support	-0.08	0.17	-0.42	0.26	-0.02	-0.44	0.66
Use instrumental support	0.26	0.16	-0.05	0.56	0.06	1.67	0.10
Behavioural disengagement	0.68	0.15	0.40	0.97	0.14	4.66	< 0.001
Venting	0.10	0.15	-0.20	0.40	0.02	0.64	0.52
Positive reframing	-0.67	0.13	-0.93	-0.41	-0.14	-5.08	< 0.001
Planning	0.27	0.15	-0.03	0.56	0.06	1.75	0.08
Humour	-0.10	0.14	-0.37	0.17	-0.02	-0.73	0.47
Acceptance	-0.21	0.15	-0.50	0.08	-0.04	-1.42	0.16
Religion	-0.09	0.09	-0.27	0.10	-0.02	-0.93	0.35
Self-blame	1.11	0.12	0.88	1.33	0.27	9.60	< 0.001

Note: Step 1 R^2^ = 0.28, F(10, 852) = 33.80, p < .001; Step 2 R^2^ = 0.50, F(24, 838) = 34.34, p < .001

### Predictors of positive mental well-being

Table 3 details the relationship between positive mental well-being and the sociodemographic variables. Positive mental well-being was significantly higher for ICs who had undergone postgraduate training compared to those who hadn’t, t(547)=-3.01, p < .01, g = 0.26, with a small effect size. Spouses/ partners had significantly lower well-being than the other relationship categories, t(861)=-2.57, p = .01, g = 0.24, with a small effect size. The following were included in step 1 of the regression: IC age; whether the IC was a spouse/partner, had post-graduate education, had received an intervention/ supports related to their caregiving role, had a chronic illness, or was living with the person with BPD; hours of contact per week with the person with BPD; COVID-19 anxiety; and perceived social support.

Table 5 summarises the regression results. The sociodemographic variables, COVID-19 anxiety, and perceived social support contributed significantly to the regression model, R^2^ = 0.22, F(9, 853) = 26.56, p < .001. Use of coping strategies added 15.8% to the predictive capacity of the model with a medium to large effect size when controlling for IC age, education, receipt of intervention, chronic illness, relationship status, cohabitation, hours of contact, COVID-19 anxiety, and perceived social support, ΔR^2^ = 0.16, ΔF(14,839) = 15.20, p < .001, f^2^ = 0.25.

**Table 5:** Hierarchical Multiple Regression Model of Predictors of Informal Carer Positive Mental Well-being

			B (95% CI)				
	B	SE B	Lower	Higher	β	t	p
Step 1							
Constant	18.76	4.63	9.68	27.84		4.06	< 0.001
Age	0.18	0.07	0.05	0.31	0.09	2.76	0.01
Education (post-graduate)	0.81	1.36	-1.85	3.47	0.02	0.60	0.55
Relationship (spouse/partner)	1.96	1.94	-1.85	5.78	0.03	1.01	0.31
Received intervention	2.95	1.51	-0.02	5.92	0.06	1.95	0.05
Chronic illness	-6.92	1.43	-9.72	-4.11	-0.15	-4.84	< 0.001
Cohabitation	-5.07	1.56	-8.14	-2.01	-0.12	-3.25	< 0.01
Hours of contact	-0.03	0.01	-0.05	0.00	-0.07	-1.86	0.06
COVID-19 anxiety	-1.16	0.22	-1.59	-0.73	-0.16	-5.31	< 0.001
Perceived social support	4.19	0.48	3.25	5.12	0.28	8.81	< 0.001
Step 2							
Constant	31.39	6.37	18.90	43.89		4.93	< 0.001
Age	0.16	0.06	0.04	0.28	0.08	2.57	0.01
Education (post-graduate)	1.28	1.24	-1.16	3.72	0.03	1.03	0.30
Relationship (spouse/partner)	3.83	1.84	0.22	7.45	0.07	2.08	0.04
Received intervention	0.39	1.41	-2.38	3.16	0.01	0.27	0.78
Chronic illness	-6.51	1.30	-9.07	-3.96	-0.14	-5.00	< 0.001
Cohabitation	-3.86	1.42	-6.65	-1.07	-0.09	-2.72	0.01
Hours of contact	-0.03	0.01	-0.06	-0.01	-0.08	-2.45	0.01
COVID-19 anxiety	-0.86	0.20	-1.25	-0.46	-0.12	-4.22	< 0.001
Perceived social support	2.70	0.51	1.70	3.70	0.18	5.29	< 0.001
Self-distraction	-0.55	0.40	-1.34	0.24	-0.04	-1.37	0.17
Active coping	0.62	0.47	-0.31	1.54	0.05	1.31	0.19
Denial	-0.83	0.56	-1.94	0.28	-0.05	-1.47	0.14
Substance use	-0.90	0.34	-1.57	-0.24	-0.08	-2.68	0.01
Use emotional support	0.59	0.52	-0.43	1.62	0.05	1.14	0.25
Use instrumental support	-0.51	0.46	-1.42	0.41	-0.04	-1.09	0.28
Behavioural disengagement	-1.56	0.44	-2.42	-0.70	-0.12	-3.55	< 0.001
Venting	-0.44	0.46	-1.35	0.47	-0.03	-0.95	0.34
Positive reframing	2.50	0.40	1.73	3.28	0.20	6.31	< 0.001
Planning	-1.38	0.45	-2.27	-0.48	-0.11	-3.02	< 0.01
Humour	1.08	0.41	0.27	1.88	0.08	2.62	0.01
Acceptance	0.93	0.44	0.07	1.80	0.07	2.12	0.03
Religion	0.49	0.28	-0.07	1.05	0.05	1.73	0.08
Self-blame	-1.68	0.34	-2.36	-1.00	-0.15	-4.87	< 0.001

Note: Step 1 R^2^ = 0.22, F(9, 853) = 26.56, p < .001; Step 2 R^2^ = 0.38, F(23, 839) = 22.06, p < .001

Age, chronic illness, and cohabitation were the only significant sociodemographic predictors of positive mental well-being. COVID-19 anxiety also negatively predicted positive mental well-being. Perceived social support and coping strategies of positive reframing, humour, and acceptance were significant positive predictors of positive mental well-being. Self-blame, behavioural disengagement, planning, and substance use were significant negative predictors of positive mental well-being. Positive reframing, perceived social support, and self-blame were the strongest predictors. They uniquely explained 3.0%, 2.1% and 1.7% of the variance in positive mental well-being, respectively. Behavioural disengagement (0.9%), planning (0.7%), substance use (0.5%), humour (0.5%) and acceptance (0.3%) each uniquely explained a minimal amount of the variance in positive mental well-being.

## Discussion

ICs reported levels of psychological distress greater than population norms, concordant with previous research [14–16]. The current research also showed that ICs experienced lower levels of positive mental well-being than population norms and other caregiving populations, thus extending understanding of carer experience beyond negative outcome measures such as burden and psychological distress. This is an important contribution to the understanding of the health of this population, as good mental health goes beyond the absence of distress and requires the presence of positive psychological well-being [50].

Consistent with the stress process model of caregiving, perceived social support and coping strategies predicted the outcome variables when controlling for sociodemographic variables and COVID-19 anxiety. Perceived social support predicted higher positive mental well-being and lower psychological distress, echoing widespread carer research [34, 45, 76, 77]. Carers attending group interventions and support programmes have previously described the positive effect of received group peer support [11, 23, 78]. Collectively, these findings suggest that multi-family group interventions for ICs may benefit from a specific focus on participants developing social supports both within and outside the treatment group.

Consistent with previous research [42, 44], positive reframing was the strongest coping predictor of better outcomes, including higher positive mental well-being and lower psychological distress. In this study, humour and acceptance coping were also positively related to positive mental well-being. However, their predictive strength was lesser, and these strategies were not significant predictors of psychological distress. Of interest, previous research has categorised humour and acceptance coping with positive reframing to form a coping category of cognitive restructuring [79]. Combined, the results suggest that assisting ICs to make sense of caregiving experiences and the caregiving relationship may help the IC to form differing cognitive appraisals of typical caregiving stressors and may improve the effectiveness of interventions and IC well-being. ICs attending a carer intervention have said that content has helped them to change their understanding of the person with BPD’s behaviour, be more accepting of the caregiving situation and the person with BPD, and to consider more positively their own needs in the caregiving relationship [78], suggesting that existing interventions may support cognitive reappraisal. However, the mechanism through which this may be achieved is not known and further research is required to establish if existing interventions assist development of cognitive reappraisal and more specifically positive reframing.

Self-blame coping was the strongest coping predictor of higher psychological distress and lower positive mental well-being, consistent with IC qualitative interviews [10, 12] and findings in ICs of people with other mental health disorders [40, 43]. This is not surprising as, although today BPD is understood to have a complex aetiology [80], historical literature emphasised the causal role of the family [81]. The current study did not assess the quality of the self-blame statements of participants. However, the results provide evidence that negative self-judgments in response to caring for a person with BPD may be linked to poorer well-being and a risk factor for adverse outcomes in this population. This finding raises questions about self-blame and relationship type. For example, is the corrosive nature of self-blame the same or experienced differently in parents as compared to partners? Exploring the nature of self-blame across, and between, relationship types may advance interventions targeting the corrosive impact of self-blame. Inclusion of accurate information about BPD and also components to directly identify and address self-critical thoughts about the carer and the caregiving situation may further enhance effectiveness of existing interventions. Indeed, existing carer interventions provide up-to-date psychoeducation about the development of BPD [18, 21, 22] and other components such as adopting a non-judgmental stance [18], which may assist in reducing IC self-blame. Further research should investigate if existing interventions support a pre- to post- reduction in self-blame and the intervention components related to any change.

Behavioural disengagement from the stressor (giving up) and substance use predicted greater psychological distress and lower positive mental well-being, although the predictive strength for substance use was less important. Denial also explained minimal variance in psychological distress but not positive mental well-being. These three strategies are often categorised as avoidant coping [82, 83]. The findings are consistent with a body of evidence showing avoidance to be associated with increased adverse outcomes in response to stressful life events, including caregiving stress [38, 48, 55].

Use of disengagement coping is related to lower perceived control, which connects to lower well-being [84]. ICs of people with BPD report feeling powerless and note a lack of knowledge about BPD and about how to respond to the person with BPD [9, 10, 12], indicating a lack of perceived control over the caregiving situation. It may be useful to consider this within the context of the expressed emotion/emotional overinvolvement literature. ICs of people with BPD report difficulties in knowing when to step in (sometimes to the level of ‘emotional overinvolvement’), and when/how to step back and allow the person to make their own choices [8]. At times, ICs mention that emotional overinvolvement (often fuelled by both love and anxiety) can lead to neglect of their own health [10, 12] and has been associated with higher carer burden [2]. On the other hand, ICs have revealed wanting to “give-up” at times [11, 13]. This suggests that some ICs may oscillate from emotion overinvolvement to a position of avoidance/ disengagement. Interventions may improve carer outcomes by supporting ICs in balancing the extremes of overinvolvement and disengagement, by increasing knowledge, skills, and prioritising their own self-care and reducing substance use. This is supported by qualitative reports that one intervention [18] helped ICs of people with BPD to clarify their role in supporting a person with BPD and increased their perceived control over their own needs and how they may more effectively respond in the caregiving relationship [78]. Further research is required to investigate whether, and if so how, current interventions facilitate reduced behavioural disengagement and whether this is related to changes in emotional overinvolvement and perceived control.

Interestingly, in this study, planning related to a reduction in positive mental well-being, though it only explained a minimal amount of variance. Planning involves identifying problem-focused strategies to respond to a stressor and is typically associated with better outcomes. Problem-focused strategies are optimal when coping with controllable stressors [79]. The uncertainty and impulsivity often associated with BPD may add challenges to IC planning. This hypothesis is supported by IC reports that they cannot make plans due to the uncertainty associated with the caregiving role [10]. In this context, interventions may benefit from assisting ICs to flexibility identify when planning is an effective coping response and when it may serve to augment distress in response to a caregiving situation.

Several sociodemographic factors predicted mental well-being, helping identify which carers may be most in need of support. The current results suggest that younger ICs and those living with or in more contact with the person with BPD may be most in need of support in addition to carers with an existing chronic illness or with higher levels of anxiety, such as COVID-19 anxiety. In this study, relationship status was significantly associated with psychological distress; however when controlling for other sociodemographic variables, including age and gender, relationship status was not a predictor. No relationship was observed between the length of the caregiving relationship and outcomes, consistent with previous studies [14, 16]. Receipt of intervention/support in relation to the caregiving role predicted lower psychological distress, but there was no relationship with positive mental well-being. This indicates that the positive effects of interventions offered may not extend to positive mental well-being, an important aspect of overall health. The analysis did not differentiate the type of intervention received, and so the finding should be considered with caution.

### Limitations

The findings must be considered in the following context. The study was cross-sectional, limiting statements of causality [85]. The sample may not be generalizable due to the majority of the sample being North American, female (85.6%), and likely support-seeking, evidenced by a large proportion who endorsed receiving an intervention/ supports (74%). The BPD diagnosis was assessed by IC self-report, verified using a screening instrument, the MSI-BPD-C. Carer adaptations of this instrument have been widely used for this purpose [2, 11, 14, 15, 23]; however it has not been validated. It is recommended that future research is undertaken to assess gender, cultural, and geographical influence on relationships studied herein and include clinical assessment of the BPD diagnosis.

## Conclusions

This study shows that ICs of people with BPD experience low levels of positive mental well-being, in addition to the high levels of distress previously reported in the literature. This study addresses a shortage of existing empirical research exploring modifiable factors associated with outcomes. It identifies that perceived social support and coping strategies are significant predictors of both psychological distress and positive mental well-being in ICs of people with BPD. Existing interventions may be enhanced by incorporating or further developing components that directly assist ICs to develop quality perceived social supports; to positively reframe cognitions; to become aware of and address self-judgments and self-blame in response to caregiving stress; to move away from coping strategies such as behavioural disengagement; and to consider when planning coping may be effective and when it may be maladaptive in response to caregiving stressors. Future research should further explore the role of ICs’ understanding of BPD, interpersonal skills, appraisals of BPD and caregiving, perceived control, and how these factors relate to the use of coping strategies and IC outcomes.

## Data Availability

The datasets used and/ or analysed during the current study are available from the corresponding author on reasonable request.

## Abbreviations

BPD: Borderline personality disorder
IC: Informal carer
EUPD: Emotionally unstable personality disorder
MSI-BPD-C: McLean Screening Instrument for Borderline Personality Disorder- Carer Version
K10: Kessler Psychological Distress scale
WHO-5: World Health Organisation- Five Well-being Index
MSPSS: Multidimensional Scale of Perceived Social Support
CAS: Coronavirus Anxiety Scale
